# Treatment length and outcomes among adolescents: a secondary data analysis

**DOI:** 10.1186/1747-597X-7-35

**Published:** 2012-08-16

**Authors:** Katherine J Riley, Priya Srikanth, Dongseok Choi, Dennis McCarty

**Affiliations:** 1 Public Health & Preventive Medicine, Oregon Health & Science University, Portland, OR, USA; 2 Public Health & Preventive Medicine, Oregon Health & Science University, Portland, OR, USA; 3 Department of Public Health & Preventive Medicine, CB669, Oregon Health & Science University, 3181 SW Sam Jackson Park Road, CB669, Portland, OR, 97239-30, USA

**Keywords:** Substance use, Adolescent treatment, Treatment length, MET/CBT-5, Evidence-based program implementation

## Abstract

**Background:**

Adaptations to evidence-based substance abuse treatment programs may impact their effectiveness. A qualitative study of MET/CBT-5 implementation in community agencies treating adolescents found that the majority of the agencies made adaptations and that the most frequent adaptation was to provide more than five treatment sessions.

**Methods:**

Baseline and outcome data from SAMHSA’s Effective Adolescent Treatment demonstration were analyzed to assess associations between length of treatment, client characteristics, and outcomes at three months.

**Results:**

Adolescents who received more or less than the protocol length of 5 sessions were less likely to be discharged to the community than those who received the 5 session protocol. Those who received more than five sessions were more likely to have higher severity scores at intake but almost 50% of those with more than five sessions had low intake severity scores. Clients who received less than five sessions tended to have lower severity scores than clients who received more than five sessions.

**Conclusions:**

Length of treatment tended to vary by site rather than severity of substance problems or frequency of use. There was no significant improvement of substance abuse problems or decrease in frequency of use with longer treatment. Implementation of the MET/CBT-5 component of the Cannabis Youth Treatment trial in the EAT project illustrates the difficulty of adherence to an evidence based protocol in the field.

## Background

Development and implementation of evidence-based treatment for substance-abusing adolescents is critical because adolescents who use drugs are more likely to suffer from dependence in their lifetime [1]. The Substance Abuse and Mental Health Services Administration (SAMHSA), Center for Substance Abuse Treatment (CSAT) sponsored the Cannabis Youth Treatment (CYT) trial [2] to test and compare five models of care. Subsequently, the Effective Adolescent Treatment (EAT) program supported replication and expansion of the most cost-effective model of care – MET/CBT-5 (two sessions of motivational effectiveness treatment (MET) plus three sessions of cognitive behavioral treatment (CBT)).

Implementation of evidence-based practices generally requires acceptance of new procedures—a change from the usual. An organization’s structure, norms, and decision-making processes can affect diffusion and characteristics of newly implemented interventions. Compatibility with current practice, complexity of the intervention, timing, communication, and the characteristics of the innovation affect implementation [3]. A review of implementation research and the difficulties faced in introducing new programs and determining their impact noted progress in increasing the use of evidence-based practices but “the science related to implementing these programs with fidelity and good outcomes for consumers lags far behind” (p. vi) [4]. Fidelity of implementation is as important for outcome as the quality of the treatment. An analysis of recidivism rates suggested that good treatment that was poorly implemented was no better than poor treatment implemented extremely well [5,6]. Programs should optimally choose the strongest intervention that they can implement well.

Implementing and evaluating an evidence-based substance abuse treatment intervention is challenging. To maintain fidelity in Multidimensional Family Therapy (MDFT), Liddle et al. provided a six-month training period with regular supervision, co-therapy sessions, and booster meetings [7,8]. Similarly, an implementation study of Functional Family Therapy (FFT) included two years of counselor training and weekly consultation using a conceptual framework of adoption of innovations with attention to the complex influences on implementation [9]. Some clinicians, however, had difficulty accepting the model and wanted to provide additional services [9].

Many counselors believe that longer treatments are required. Based on previous research, the National Institute of Drug Abuse (NIDA) recommended at least 90 days of treatment to support stable recovery [10]. In contrast to these recommendations, treatment for adolescents has a history of limited duration because more than 50% of adolescents drop out of treatment within 6 weeks [11].

The limited contact of adolescents with treatment was recognized in the design of the Cannabis Youth Treatment (CYT) randomized clinical trial. The study tested five adolescent treatment programs of varying lengths. In addition to four 12–13 week programs (Family Support Network [FSN], Adolescent Community Reinforcement Approach [A-CRA], MDFT, and MET/CBT-12), brief (6–7 week) outpatient treatment of adolescents was included because over 75% of adolescents receive that much or less in the public treatment system [12]. The five session MET/CBT-5 substance abuse treatment model had results similar to the other four programs and was one of the most cost-effective and cost-beneficial models [12,13].

CSAT selected MET/CBT-5 for a post randomization replication study in 38 Effective Adolescent Treatment (EAT) awards. CSAT and the National Institute on Alcohol Abuse and Alcoholism (NIAAA) supported a qualitative study of implementation in nine EAT sites. In depth, quarterly interviews of staff at 9 sites indicated that although agency counselors and supervisors were trained and certified in the manualized MET/CBT-5 protocol, almost all sites made adaptations [14]. Some modified the protocol to address individual client needs such as multiple drug use, streetwise clients, ethnic subcultures, and gender differences. One agency added psychiatric sessions for clients with a co-occurring diagnosis of mental health issues. Additional adaptations were made to adjust to agency and client pressures, including delivery of the CBT portion on an individual basis to avoid scheduling conflicts or transportation barriers and the inability to bring clients together in groups. Several agency representatives reported that onsite supervision sessions gradually declined in frequency because the counselor or the supervisor was busy or had confidence in the counselor’s ability to perform as trained [14]. The most frequent adaptation was the addition of more treatment sessions, sometimes in response to parental or referring agency requests for more treatment [14]. The EAT clients had a larger proportion of minority clients than the CYT sample, were less likely to have their primary substance as marijuana, and had a greater range of substance abuse and mental health issues but the mean levels of severity were similar to the CYT study [15].

The current study used data that were available from EAT sites to assess relationships between treatment length, client characteristics and treatment outcomes. Two research questions were examined:

● What were the characteristics of clients who received more treatment and do the differences indicate that more treatment was needed for those who received it?

● Did the clients who received longer treatment have better outcomes than those who had the protocol length of treatment?

## Methods

### Data source

Clients enrolled in EAT programs completed the Global Appraisal of Individual Needs (GAIN) [16] to record intake status and to assess change over time. The GAIN includes multiple assessment instruments [16] and has been used in varied settings and projects [2,17]. The GAIN-I, the initial assessment, was administered by counselors that received either one week of training off-site or received training on-site from certified local trainers. Clients provided self-reports to the counselors in 50–120 minute individual sessions. Counselors submitted audiotapes from their agency sessions for trainer review and certification of counselor competency. The counselors also received weekly supervision from agency staff who were trained and certified in the GAIN and in GAIN supervision. The GAIN-M90 (a shorter, monitoring version) was administered in the same individual manner by a counselor 90 days after intake.

The GAIN TTL (Treatment Transition Log) documented the date of admission and discharge for each level of care (e.g., outpatient, intensive outpatient, short term residential), including prior level of care, referral source, current level of care, the type of treatment received (i.e., specific manualized interventions) and discharge status. Discharge and admission dates were subtracted to calculate treatment duration [18].

The GAIN 2007 dataset of client level data from the EAT cohort was used for this study. Permission was requested of the EAT grantees to use their data in a de-identified dataset. The Oregon Health & Science University’s Human Subjects Institutional Review Board determined the secondary analysis met criteria for exemption. Usable data were available from 36 sites with 6,527 clients who had participated in follow-up assessments 3 months after intake. Clients who were assessed at the 3- and 6-month follow up interviews were compared to determine whether those who did not complete the 6-month follow up (n = 618) were significantly different than those who completed it. Clients who were not available for the 6-month follow-up tended to be older than the predominant group of 14–17 year olds (χ_2_^2^ = 24.14); no other differences were significant. Based on the lack of major differences and the desire to have the largest number of clients for analysis, the 3- month follow-up group was selected for further analysis.

**Figure 1:**
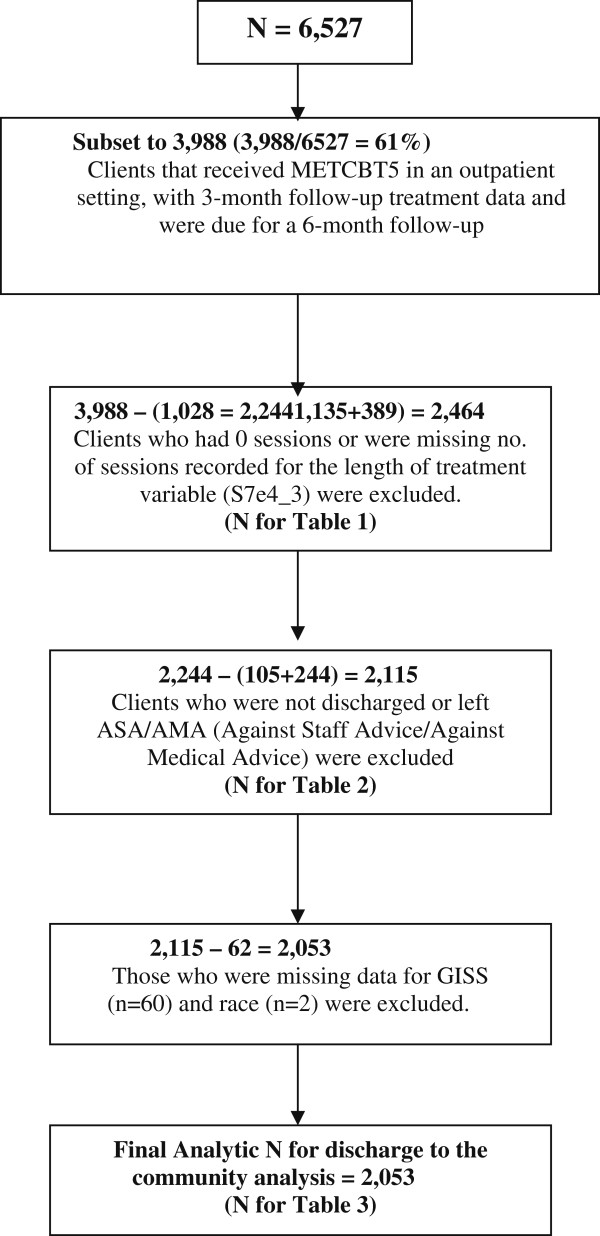
Client exclusion data analysis flow chart.

Clients were excluded if they did not receive METCBT5 in an outpatient setting (n = 79), were not in outpatient treatment (n = 392), had no record of EAT treatment at 3 months (n = 749), and did not have a 3-month follow-up record (n = 2,187), leaving a total of 3,988 clients. Clients who had 0 sessions recorded for the length of treatment (N = 1,135) or were missing the length of treatment (N = 389) were excluded from the analysis. Also excluded were those who were still in treatment (n = 105) and those who left against staff advice (ASA) or against medical advice (AMA) (n = 244). Those with missing data for the General Individual Severity Scale (GISS) (n = 60) and race (n = 2) were also excluded. These exclusions further reduced the sample size to 2,053 clients at 34 sites. See Figure 1. Final sample size used for each analysis is listed in the relevant sections.

### Key variables

Demographic characteristics included gender, age, race/ethnicity, current criminal justice involvement, and primary substance used.

Client severity: Clients who entered treatment with higher scores on measures of behavior, mental, substance frequency use, and/or substance problems at intake baseline were considered to potentially have more difficulty in responding positively to substance abuse treatment than clients with lower scores. The General Individual Severity Scale (GISS) assessed problem severity [19] by counting symptoms across 15 scales (123 items, coded as 0-28 = 0, 29-50 = 1, 51-123 = 2 with higher scores indicating greater severity; alpha of .97 in adolescents): The items form four dimensions:

*Behavior Complexity Scale (BCS)*: (alpha = .94, mean 10.57, sd = 8.363) BCS scores range from 0 to 33. BCS scale cutoff scores were categorized as 0–5, 6–18, and 19–33. A count of external behavioral problems, such as fidgeting, being forgetful, being disorganized, avoiding things that took too much effort, starting fights, setting fires, vandalism, running away, etc., reported in the past year. Higher scores represent increasing difficulty controlling external behavior (e.g., ADHD, Conduct Disorder).

*Internal Mental Distress Scale (IMDS)*: (alpha = .94, mean = 8.28, sd = 8.785) IMDS scores range from 0 to 43. Cutoff scores were categorized as 0–8, 9–23, and 24–43. Count of symptoms including somatic, anxiety, depression, traumatic stress and suicide/homicide. Higher scores are associated with risk for suicide and violence.

*Substance Frequency Scale (SFS8p- abbreviated to SFS for this study):* (alpha = .79, mean = .12, sd = .14). Cutoff scores were categorized as 0, 0.01-0.13, and 0.14-1.00. Average percent of days (of the past 90) reported for any alcohol or other drug (AOD) use; heavy AOD use; problems from AOD use; and days of alcohol, marijuana, crack/cocaine, heroin/opioid, and other drug use. Higher scores represent increasing frequencies of substance use in terms of days, days staying high most of the day (i.e., high risk of problems), and days actually causing problems. People with scores over .14 may have considerable difficulty stopping without significant assistance and/or a controlled environment.

*Substance Problem Scale* - Past Month (SPSM): (alpha = .89, mean = 2.58, sd = 3.492) Scores range from 0–16. The cutoff scores were categorized as 0, 1–9, and 10–16. Three subscales were examined: Substance Issues Index in the past month [SIIM], Substance Abuse Index in the past month [SAIM], and Substance Dependence Scale in the past month [SDSM]). The SIIM includes a count of symptoms of substance related problems including two for substance-induced health and psychological problems, and three on lower severity symptoms of use (hiding use, people complaining about use, weekly use) that a client reports having in the past month. The SAIM includes a count of symptoms of substance abuse in the past month that were endorsed by the client. The SDSM includes a count of symptoms of substance dependence in the past month that were endorsed by the client. Interpretation: Higher SPSM scores represent greater severity of drug problems. The scale includes physiological, psychological, and social criteria, as well as an item on co-morbid use with drugs that is likely to exacerbate the other problems. It is associated with increased odds of externalizing and internalizing problems.

*Crime/Violence Scale (CVS)*: (alpha = 0.90, mean = 3.128, sd = 5.987) The cutoff scores were categorized as 0–2, 3–6, and 7–31. Count of strategies used during the past year to resolve interpersonal conflict and types of property, drug related, and interpersonal crimes committed. The scale is designed to predict future crime and violence [19,20]. This measure was only included on the initial GAIN. Higher values indicate greater involvement in illegal activities, and/or more violent strategies.

Clients with low GISS scores (0) are unlikely to need services. Clients with Moderate scores (1) and who have a possible diagnosis are likely to benefit from a brief intervention. Higher GISS scores (2) have a high probability of diagnosis and need more formal assessment and intervention. High scores are indicative of more severe problems in substance use, mental and physical health, and illegal activities [19,20]. (A more detailed description of these and other GAIN scales can be obtained at http://www.chestnut.org/LI/gain/index.html#supporting%20psychometrics,%20scales,%20and%20crosswalks). The GISS scale was administered at intake and subscales were included in the M90 assessment 90 days after intake.

Length of treatment: The MET/CBT-5 protocol specifies five sessions of care. The variable ‘how many times did you go to a regular outpatient program’ (s7e4_3) was used to code the number of sessions based on the client’s self report. To designate adherence to the protocol length, the number of sessions was coded as less than five sessions (1–4), five sessions, and greater than 5 sessions.

Sites: The CYT study found that site effects were important [12] In the qualitative analysis, EAT sites also appeared to differ based upon offering a predominant pattern of treatment length [14]. Site characteristics were considered as possible influences on length of treatment; e.g., staff culture emphasizing longer treatment, reimbursement per session, or predominant characteristics of clients could influence counselors to add sessions. Available site information was reviewed to determine if there were any different patterns. Sites were provided with a flat payment rather than payment per client visit and counselor attitudes and site culture were not measured in the study. To control for possible unmeasured influences, therefore, the 34 sites were included as a nominal variable in the multinomial logistic regression and as a randomized effect in the multi-level mixed-effects logistic regression modeling.

Outcome measures:

Discharge Status: Completed clients were dichotomized based on discharge destination. Discharge to the community was considered the optimum treatment result. All other discharge categories required continuing treatment or justice involvement and were combined.

Substance problems: Self report of substance abuse problems and substance abuse frequency at 3-month follow-up were used to determine whether clients were still experiencing problems with substance use after or close to completion of treatment. Because the GISS scale was only administered at intake, the SFS and SPSM subscales at intake were compared with their 3-month follow up scores to determine if changes in usage or problems might be an indicator of outcome success. The SFS scale provides information on average percentage of days reported of alcohol or drug use in the past 90 days. The SPSM scale provides an indicator of substance problems. Both scales can also be used to compare substance activities at two different points in time. The variables SPSM and SFS at intake, described in the GISS variable section, were compared with scores on these variables at 3-month follow up to determine whether substance use and frequency had increased, remained the same, or decreased after that time period had elapsed and treatment had been completed to see whether those outcomes were associated with discharge status and length of treatment.

### Data analysis

Characteristics of excluded clients were compared with the final sample to determine if there were significant differences using Pearson’s *χ*^2^ test. For the final sample contingency tables and Pearson’s *χ*^2^ test were used to examine the associations between client characteristics and length of treatment (5 sessions, <5 sessions, and >5 sessions). Client characteristics included baseline age, race, gender, primary substance used, GISS scores at intake, baseline scores of 5 subscales (IMDS, BCS, SFS, and SPSM), 3-month scores of 2 subscales (SFS and SPSM), and criminal justice involvement at baseline. ANOVA was used to test whether the mean of the continuous variable CVI differed by length of treatment. Associations were also examined between discharge status and client characteristics that included treatment length, gender, race, baseline age, GISS, and three month SPSM and SFS scores at three-month follow up. In these analyses SPSM and SFS were used as dependent variables.

A multi-level mixed effects logistic regression model [21] was used to test whether treatment length was associated with discharge to the community after adjusting for client characteristics. Client characteristics included baseline age, race, gender, GISS at intake, 3-month SPSM and SFS scores, and site. Site was treated as a random effect to accommodate for the varying baseline covariates at each site. To assess for colinearity, correlation was checked using Pearson’s correlation coefficient, between the 3-month SPSM scores, 3-month SFS scores, and the length of treatment variable.

To assess whether treatment length was associated with substance use outcomes at 3 months after adjusting for other covariates, a multinomial logistic regression model was used. Separate models for 3-month SPSM scores and 3-month SFS scores were run with the same set of adjustments in each model. Adjustments included baseline age, race, gender, and GISS at intake. All 34 sites were included as a nominal variable. All data analyses were conducted using STATA 11.0 [22].

## Results

### Descriptive analysis- client characteristics

In comparing the final sample with clients who were excluded from the analysis, the final sample tended to be younger (X_2_^2^ = 12.352, p = .002) and clients were more likely to be white than minority (X_1_^2^ = 20.93, p < .001). In addition, their primary abuse substance was more likely to be alcohol or marijuana whereas those who were excluded were more likely to use other drugs (X_3_^2^ = 17.9159, p < .001). There were also differences in problem severity with clients in the final sample having moderate baseline severity scores in comparison with excluded clients who tended to have lower overall scores (X_2_^2^ = 12.1696, p = .002), including moderate baseline behaviorial conflict (X_2_^2^ = 32.1688, p < .001) and substance use frequency scores (X_2_^2^ = 10.6907, p = .005) in comparison with lower scores for excluded clients. At three month follow-up the final sample had lower substance use frequency scores and those who were excluded had higher frequency of usage (X_2_^2^ = 17.3637, p < .001). There were no differences in gender, criminal justice involvement, baseline internal mental distress and substance problems, or three month substance problems.

In the final sample at 3-month follow-up, almost half of the discharged adolescents had received more than 5 sessions (45%), one-third received 5 sessions (34%), and almost a quarter (22%) received less than 5 sessions. See Table 1. Younger (χ_4_^2^ = 14.83, p = .005), non-white (χ_2_^2^ = 7.46, p = .024) clients were more likely to receive less than 5 sessions. Clients who had criminal justice involvement at intake were more likely to receive additional treatment than the protocol number of sessions (χ_2_^2^ = 38.12, p < .001). Clients who used marijuana were more likely to receive 5 sessions or less and clients using “other drugs”, including inhalants and narcotics, were more likely to receive additional sessions (χ_2_^2^ =45.81, p < .001). An examination of site characteristics for sites that predominantly provided less than 5 sessions, 5 sessions, or more than 5 sessions determined that there were no patterns that differentiated one group from another in terms of population or racial composition of their cities or other characteristics. However, the 6 sites that offered predominantly fewer sessions to 60% of their clients tended to have a small number of clients and averaged only 31 clients per site and 3 of the sites had not provided the 5 session protocol to anyone, including two sites that only had one client each. In contrast, 11 sites predominantly provided the 5 session protocol and 17 sites predominantly provided more than 5 sessions. The average number of clients served at those sites was similar for both of these two groups; 76 and 68 respectively. However, 70% of the clients in the first group predominantly received the protocol length of treatment whereas 72% of the clients in the last group received more than 5 sessions.

**Table 1:** EAT demographics by treatment length

	**<5 sessions**	**5 sessions**	**>5 sessions**	**Total**	**chi-square**
Gender	N = 544	840	1,080	2,464	χ_2_^2^ = 2.93
Male	66%	69%	70%	69%	p = 0.231
Female	34%	31%	30%	31%	
Age @ baseline	N = 544	840	1,080	2,464	χ_4_^2^ = 14.83**
10-14	28%	23%	21%	24%	p = 0.005
15-17	63%	70%	71%	68%	
18-22	8%	7%	8%	8%	
Race/Ethnicity	N = 543	839	1,078	2,460	χ_2_^2^ =7.46**
Whites	45%	43%	49%	46%	P = 0.024
Non-Whites	55%	57%	51%	54%	
Current Criminal Justice Involvement	N = 544	839	1,079	2,462	χ_2_^2^ = 38.12**
Yes	58%	58%	70%	63%	p < 0.0001
Primary substance based on symptom severity	N = 544	840	1,080	2,464	χ_6_^2^ = 45.81**
Alcohol	12%	17%	14%	14%	p < 0.0001
Amphetamines	11%	11%	6%	9%	
Marijuana	41%	38%	35%	37%	
Other Drug	36%	33%	45%	39%	
General Individual Severity Scale	N = 531	821	1,039	2,391	χ_4_^2^ = 24.63**
Low - 0	53%	57%	47%	52%	p < 0.0001
Moderate - 1-2	29%	30%	34%	32%	
High > =3	19%	13%	19%	16%	
Crime Violence Index	N = 544	839	1,078	2,461	F_(2,2458)_ = 7.72**
	6.3 (±5.1)	5.7 (±4.6)	6.6 (±5.1)	6.2 (±5.0)	p = 0.0005
Internal Mental Distress Scale	N = 544	839	1,079	2,462	χ_4_^2^ = 17.82**
0-8	65%	71%	62%	66%	p < 0.001
9-23	27%	24%	31%	28%	
24-44	8%	5%	7%	7%	
Behavioral Complexity Scale	N = 544	837	1,078	2,459	χ_4_^2^ = 20.19**
0-5	34%	37%	30%	33%	p<0.0001
6-18	48%	51%	54%	52%	
19-33	18%	12%	16%	15%	
Substance Problem Scale Past Month	N = 544	839	1,079	2,462	χ_4_^2^ = 13.47**
0	40%	39%	34%	37%	p = 0.009
1-9	53%	57%	59%	57%	
10-16	8%	4%	7%	6%	
Substance Frequency Scale	N = 544	840	1,080	2,464	χ_4_^2^ = 40.29**
0	16%	17%	12%	14%	p < 0.0001
0.01-0.13	53%	60%	52%	55%	
0.14-1.00	31%	23%	36%	31%	
Substance Problem Scale Past Month (3-months)	N = 543	839	1,079	2,461	χ_4_^2^ = 13.46**
0	64%	59%	57%	60%	p = 0.009
1-9	34%	36%	40%	37%	
10-16	2%	4%	4%	3%	
Substance Frequency Scale (3-months)	N = 544	840	1,080	2,464	χ_4_^2^ = 21.49**
0	31%	35%	32%	33%	p < 0.0001
0.01-0.13	50%	54%	51%	52%	
0.14-1.00	19%	11%	17%	15%	

** significant at the 0.01 level.

*significant at the 0.05 level.

Associations between baseline GISS scores and length of treatment indicated that most clients (52%) had low severity scores at intake. Adolescents with higher severity scores were just as likely to receive more or less than five sessions and those with low scores were more likely to receive five sessions or less than the recommended number of sessions (χ_4_^2^ = 24.63, p < .001). Clients with moderate severity scores were slightly more likely to be associated with receiving 5 sessions or more. Significant associations with length of treatment were also found for the component GISS scales of Crime Violence (F_2, 2458_ = 7.72, p < 0.001), Internal Mental Distress (χ_2_^2^ =17.82, p < .001), Behavioral Complexity (χ_2_^2^ =20.19, p < .001), and SPSM (χ_2_^2^ =13.47, p < .001), although most clients had moderate or low scores. Most clients had higher scores on the SFS scale and those with the highest scores were more likely to receive more sessions (χ_2_^2^ =40.29, p < .001). However, many clients in the group of high or moderate scorers for this variable received less than the protocol number of sessions.

Substance problems and frequency of use at 3 months after intake were significantly associated with number of sessions received. Increased substance problems (χ_4_^2^ = 13.46, p = .009) was associated with receiving more treatment sessions, although there were still many in the high scoring category that received fewer sessions than the protocol length of five sessions. Increased frequency of use was associated with both receiving less than and more than the protocol number of sessions (χ_4_^2^ = 21.49, p < .001).

### Discharge outcomes and length of treatment

Length of treatment was associated with discharge destination among the completed EAT clients (χ_2_^2^ = 139.53, p < .001). See Table 2. Overall, a majority of clients were discharged into the community (81%; 1,710 of 2,115). Of the clients who received the protocol length of treatment (5 sessions), 93% were discharged into the community, compared to 82% of those with less than 5 sessions of treatment and 70% of those who received more than 5 sessions. Race/ethnicity was not associated with discharge to the community but gender was significantly related and males were more likely to be discharged to the community than females (χ_1_^2^ = 5.03, p = .025).

**Table 2:** EAT discharge status by baseline characteristics

**Characteristic**	**Community**	**Other**	**Total**	**Chi-square**
No. of sessions				χ_2_^2^ = 139.53**
	N = 1,710	405	2,115	p < 0.0001
<5 sessions	82%	18%		
5 sessions	93%	7%		
>5 sessions	70%	30%		
Gender	N = 1,710	405	2,115	χ_1_^2^ = 5.03*
Male	82%	18%		p = 0.025
Female	78%	22%		
Race/Ethnicity	N = 1,708	405	2,113	χ_1_^2^ = 0.74
Non-Whites	80%	20%		p = 0.389
Whites	82%	18%		
Age @ baseline	N = 1,710	405	2,115	χ_2_^2^ = 10.75**
10-14	85%	15%		p = 0.005
15-17	79%	21%		
18-22	85%	15%		
GISS				χ_2_^2^ = 29.98**
	N = 1,665	390	2,055	p < 0.0001
Low - 0	84%	16%		
Moderate - 1-2	81%	19%		
High - > =3	71%	29%		

** significant at the 0.01 level.

*significant at the 0.05 level.

105 who were not discharged and 244 ASA/AMA were excluded for this analysis.

Clients who were discharged into the community were more likely to be in the youngest and oldest groups (10–14 or 18–22; χ_2_^2^ = 10.75, p = .005) and have lower GISS scores (χ_2_^2^ = 29.98, p < .001). At 3 month follow up there were no significant differences on SPSM or SFS scales between intake and 3 month follow up for completed clients who were discharged to the community or transferred elsewhere.

In the multi-level mixed-effects logistic regression modeling that adjusted for baseline intake scores, gender, race/ethnicity, age, site differences (as a random effect), and substance frequency at three months, the number of sessions clients received was a significant predictor of being discharged into the community (χ_2_^2^ = 25.13; p < .001). See Table 3. Clients who received more than 5 sessions of treatment were less likely to be discharged into the community compared to those who completed the protocol number of sessions (OR: 0.35, 95% CI: 0.22 - 0.53). Clients who received less than 5 sessions were also less likely to be discharged into the community (OR: 0.37, 95% CI: 0.23 – 0.60) compared to those who completed the protocol number of sessions. Gender and age did not significantly affect discharge outcomes but whites were more likely to be discharged to the community (χ_1_^2^ = 7.13, p = .007; OR: 1.52, 95% CI: 1.12-2.08)). Baseline GISS scores were significantly associated with clients being discharged to the community. Clients with high baseline GISS scores were less likely to be discharged into the community than clients with moderate or high severe scores (χ_2_^2^ = 12.31, p = .002; OR: 0.90, 99% CI: 0.65 – 1.24 and OR: 0.50, 99% CI: 0.34-0.74). When colinearity was tested between three month follow-up substance problem and substance frequency scores, the correlation coefficient was 0.65, indicating some colinearity. The three-month SPSM score was not significant, therefore, it was excluded from the final model. At three month follow up higher substance frequency scores were significantly related to discharge destination (χ_2_^2^ = 27.05, p < .001). Clients with the highest scores were less likely to be discharged to the community (OR: 0.31, 99% CI: 0.20 – 0.48).

**Table 3:** **Logistic regression results**^**+ **^**outcome: discharged into the community at 3-months (Analytic N = 2,053)**

**Variable**	**Adjusted OR (95% CI)**	**Chi-square**
No. of sessions		χ_2_^2^ = 25.13**
5 sessions	Referent	p = <0.0001
<5 sessions	0.37 (0.23, 0.60)	
>5 sessions	0.35 (0.22, 0.53)	
Race/Ethnicity		χ_1_^2^ = 7.13*
		p = 0.0076
Non-Whites	Referent	
Whites	1.52 (1.12, 2.08)	
Gender		χ_1_^2^ = 0.52
		p = 0.4688
Male	Referent	0
Female	0.89 (0.66, 1.21)	
Age @ baseline		χ_2_^2^ = 0.13
		p = 0.9393
10-14	Referent	7
15-17	1.00 (0.70, 1.43)	
18-22	1.11 (0.57, 2.13)	
GISS Scores		χ_2_^2^ = 12.31**
		p = 0.0021
Low - 0	Referent	0
Moderate - 1	0.90 (0.65, 1.24)	
High - 2	0.50 (0.34, 0.74)	
Substance Frequency Scale (3 months)		χ_2_^2^ = 27.05**
		p < 0.0001
0	Referent	
0.01 – 0.13	0.62 (0.44 – 0.86)	
0.13 – 1.00	0.31 (0.20 – 0.48)	

** significant at the 0.01 level.

*significant at the 0.05 level.

105 who were not discharged and 244 ASA/AMA were excluded for this analysis.

Mixed effects multi-level logistic regression model included covariates of race/ethnicity, gender, age at baseline, GISS scores, and SITE as a random effect.

When the effect of length of treatment on substance abuse problems (SPSM) and average frequency of use (SFS) was tested using multinomial logistic regression, no significant effects were found for either variable. Number of sessions was not a significant predictor of three month substance abuse problems or frequency of use after adjusting for baseline age, race, site, gender, and severity score (GISS) at intake. Among those who had more than 5 sessions, the risk of having a moderate SPSM score at 3-months compared to 0 SPSM was 1.71 (95% CI: 0.75-3.89). Similarly, the relative risk ratio of having a high SPSM score at three months relative to 0 SPSM was 0.92 (95% CI: 0.43-1.98). In looking at the outcomes for substance abuse frequency, for the clients who had less than 5 sessions, the relative risk ratio of having a moderate SFS score at three months relative to 0 SFS was 0.99 (95% CI: 0.72-1.35). Similarly, the relative risk ratio of having a high SFS score at three months relative to 0 SFS was 0.98 (95% CI: 0.85-2.11). For those who had more than 5 sessions, the relative risk ratio of having a moderate SFS score at three months relative to 0 SFS was 1.34 (95% CI: 0.85-2.11) Similarly, the relative risk ratio of having a high SFS score at three months relative to 0 SFS was 1.12 (95% CI: 0.72-1.75). But, these associations were not significant.

Of the 1,703 (among the 2,053) clients who were discharged and received the 3 month follow up, approximately two-thirds (65%) reported some substance use on the SFS scale. Clients who had no indication of either substance problems or substance use for both scales comprised only 33% (n = 685) of the group.

## Discussion

A large portion of participants (n = 1,080 of 2,464; 44%) received more treatment than the protocol specified. Approximately 47% of the clients who received more than the recommended length of treatment had low severity GISS scores at baseline. Most clients (52%) had low severity scores. In addition, when measures of substance problems and frequency of use at three month follow-up were tested as outcome variables in multinomial logistic regression analyses that adjusted for possible confounding of other variables there was no relationship to number of treatment sessions received. A large proportion of clients whose level of severity was rated moderate or low both at baseline and at three month follow-up reported receiving more treatment sessions than the number recommended for brief treatment under the MET/CBT-5 protocol.

Discharge into the community was viewed as an indicator of a positive treatment result. Although most clients were discharged into the community, discharge patterns do not appear to be related to substance problems at three month follow-up but greater frequency of substance use at three month follow-up was significantly related to less likelihood of being discharged to the community. If discharge destination for additional treatment was related to problem severity, the destination would be appropriate and this significant relationship between substance frequency (SFS) scores at three month follow-up for these destinations is an indicator of appropriate placement. Gender and age were not associated with discharge destination. Whites were more likely to be discharged to the community. The number of sessions received, frequency of use at three month follow-up, and severity of problems at baseline had the largest impact on discharge to the community. Although sites tended to provide more sessions to clients with higher initial severity scores and more frequent use three months after intake, they also routinely provided less or more than the protocol number of sessions to clients with low and moderate initial severity scores. There was no significant difference in discharge of their clients to the community based upon the increase or decrease of substance problems at 3 month follow-up. Many clients received less than the protocol number of sessions and although most clients in the sample (n = 2,115; 86%) were classified as having completed treatment, some of this group (n = 405; 19%) were transferred to additional treatment or judicial placement.

Another indicator of a positive outcome for treatment is a decrease in substance severity. A decrease in substance problems and frequency of use at three month follow up should be related to more treatment for those with greater problems. However, when a multinomial logistic regression model controlled for race, age, gender, baseline severity scores, and site, neither greater substance problems nor frequency of use were significantly related to length of treatment.

### Limitations

The study is a secondary analysis of data from the EAT demonstration project implementing MET/CBT-5 in a community setting. In the implementation many sites added sessions beyond the MET/CBT-5 protocol. The addition of sessions for many clients in this study differs from the CYT clinical trial and demographic differences between the samples could be a contributor to the results with the EAT clients having a larger proportion of minority and a smaller proportion of marijuana-affected clients. However, the demographic differences were largely due to the intent to include diverse populations in the EAT group to provide insight into community applications. Client characteristics were controlled in the data analyses to account for possible confounding influences.

Clients in the final dataset tended to be younger than those who were excluded, more likely to be white than minority, and more likely to have moderate as opposed to low substance use severity. These differences would be expected to increase the association of longer treatment with substance use problems and frequency of results at three month follow-up. However, the testing of these relationships did not produce significant results.

Site characteristics could also influence the length of treatment. A cultural emphasis on increasing visits to maximize payments or staff beliefs that offering more treatment is the best practice could lead to adding sessions for clients. Different racial, socio-economic, or judicial conditions of clients might also be influential. It was not possible to determine these characteristics; therefore, site was treated as a nominal variable in the logistic regression analysis and included in the multivariate analysis as a random effect. Clients are nested within sites and the mixed-effects analyses controlled for the nesting.

The dataset did not include the number of sessions received for many clients. The length of treatment/number of sessions was supplied by client self-report and could not be verified with attendance logs. Clients may not have been able to remember exact visits or distinguish between treatment sessions and other visits. However, their reports are a good proxy for their perception of the number of sessions received and client reports have been shown to be fairly accurate in other studies [23,24]. In the qualitative study it was found that some sessions had to be split due to time pressures in schools or individual needs. This could have added additional sessions. Some sessions may have lasted longer than suggested in the protocol or they could have been shorter. It was known that one site requested permission to add additional sessions for dual diagnosis clients; however, eliminating data from this site would not have accounted for enough clients to affect the analysis. In addition, it is possible that some clients who were classified as “completed” did not receive a final session until after the 3 month follow-up. The significant relationship in the multinomial logistic regression analysis of frequency of use at three month follow-up to discharge to additional care or judicial placement was an indicator that substance use severity may have been influential in the addition of sessions; therefore, additional multinomial logistic regression analyses tested for substance use severity as an outcome variable. When substance problems and frequency of use were included as predictors of treatment length, no significant relationship was found for either variable to number of sessions received. These results are further indicators of the lack of relationship between substance use and the length of treatment clients received.

Although clients who left treatment against advice had similar characteristics to the overall sample, they included a significantly lower proportion with criminal justice involvement and it is possible that they had less need for additional treatment. The dataset also does not include information on additional services clients might have received, such as parent sessions, etc.

GAIN assessments are based on client self-reports; however, cross-validation studies with client drug testing have indicated a high degree of accuracy for self reports [23]. In a study comparing adolescents entering residential treatment who had been tested with the GAIN, their self reports were largely consistent (kappa = .53 to .69) with family reports and urine testing on site [24].

The use of discharge status as a measure of outcome success provides a limited view of treatment success. Given the chronic nature of substance use, it is probable that many clients who were discharged to the community might need additional treatment in the future. Comparing the changes in client responses to the SFS and SPSM scales between intake and 3 month follow up and testing these scales as dependent variables in multinomial logistic regression analyses and as independent variables in the discharge analysis were mechanisms to minimize that effect.

Although these limitations should be considered for future research and indicate the need for replication of the results, the dataset is large and results are consistent with previous studies that indicate the effectiveness of brief treatment [17]. The strength of having client data from 34 sites spread across the United States in diverse geographic and cultural settings lends weight to the findings.

## Conclusions

The challenge of implementing an evidence-based practice in a community setting is substantial and this study provides an indication of areas that need particular attention. Findings indicate that adding sessions was not necessary for many clients and may have added cost to treatment.

The length of treatment that clients received did not appear to be based on client needs for the majority of the sites. As the qualitative study [14] suggested, site norms may have a larger impact on implementation than a manualized protocol [14,25-27]. For the sites in this study that provided more than 5 sessions to 72% of their clients, the decision to provide more sessions seems to have been made at the site level, irrespective of the instructions in their manuals and their training. If there are beliefs that more treatment is better and more reimbursement is provided for additional sessions, it is likely that clients will receive more sessions. The results of this study suggest that use of the GISS scale to determine the potential need for additional sessions might be a helpful screening tool.

Monitoring implementation needs to be ongoing; otherwise, community implementation will not replicate the results obtained in randomized clinical trials. Monitoring is tricky; however, since the use of supervisory time has associated cost and there is a tendency for supervision to decrease over time [14]. Can less expensive methods be used; e.g., web-based checklists, self-reports, peer reviews of audiotapes?

Experienced counselors may become bored with manualized treatment and they may stray from protocols [14]. Changes to provide cultural sensitivity can create a positive atmosphere for client change [28]. On the other hand, we also know that evidence-based manualized interventions obtain better results than “business as usual” [26].

How far can treatment of individual needs of staff and clients stretch the protocol without harming the outcomes? Although an innovation may not be implemented with fidelity, we do not know which adaptations are harmless in affecting outcomes and which ones are critical to effective intervention with adolescents. Without segmenting and testing the components of an intervention, it will not be possible to determine which ones have an effect on outcomes and which ones are more effective with certain types of clients.

More research is needed to identify the key variables to implement evidence-based models of treatment for alcohol and drug use disorders. Research also needs to identify protocol variables that can be modified without interfering with therapeutic outcomes. These efforts will require increased funding for empirical research on substance abuse treatment.

## Abbreviations

A-CRA: Adolescent Community Reinforcement Approach; ADHD: Attention deficit hyperactivity disorder; AMA: Against medical advice; ASA: Against staff advice; BCS: Behavior Complexity Scale; CBT: Cognitive behavior therapy; CSAT: Center for Substance Abuse Treatment; CVS: Crime/Violence Scale; CYT: Cannabis Youth Treatment randomized clinical trial that tested 5 adolescent treatment protocols; EAT: Effective Adolescent Treatment, replication study of MET/CBT-5; EBP: Evidence based problems; FFT: Functional Family Therapy; FSN: Family Support Network; GAIN: Global Appraisal of Individual Needs a tool for global biopsychosocial assessment.; GISS: General Individual Severity Scale; IMDS: Internal Mental Distress Scale; M90: GAIN modified assessment personally administered by a counselor to a client 90 days after intake.; MDFT: Multidimensional Family Therapy; MET: Motivational Effectiveness Therapy; MET/CBT-5: Motivational Effectiveness Therapy in two sessions followed by three sessions of Cognitive Behavior Therapy; NIAAA: National Institute of Alcohol Abuse and Alcoholism; NIDA: National Institute of Drug Abuse; SAIM: Substance Abuse Index – Past Month; SAMHSA: Substance Abuse and Mental Health Services Administration; SDSM: Substance Dependence Scale – Past Month; SFS: Substance Frequency Scale; SIIM: Substance Issues Index; SPSM: Substance Problem Scale – Past Month; TTL: Treatment Transition Log includes date of admission and discharge.

## Competing interests

The authors do not have any competing interests.

## Authors’ contributions

KR had lead responsibility for study design, interface with partnering agencies, conducting analysis, and coordinated manuscript development. PS and DC provided statistical analysis and PS, DC, and DM provided editorial review and comments. All authors read and approved the final manuscript.
